# Diagnosing ectopic pregnancy using the bayes theorem and neural network: a validation of a retrospective cohort study

**DOI:** 10.61622/rbgo/2026rbgo12

**Published:** 2026-04-17

**Authors:** Larissa Maroni, Pedro Clarindo Silva, Rafael Kunst, Ricardo Francalacci Savaris

**Affiliations:** 1 Hospital de Clínicas de Porto Alegre Porto Alegre RS Brazil Hospital de Clínicas de Porto Alegre, Porto Alegre, RS, Brazil.; 2 Universidade do Vale do Rio dos Sinos São Leopoldo RS Brazil Universidade do Vale do Rio dos Sinos, São Leopoldo, RS, Brazil.; 3 Universidade Federal do Rio Grande do Sul Departamento de Ginecologia e Obstetrícia Porto Alegre RS Brazil Departamento de Ginecologia e Obstetrícia, Universidade Federal do Rio Grande do Sul, Porto Alegre, RS, Brazil.

**Keywords:** Bayes theorem, Data accuracy, Diagnosis, Ultrasonography, Ectopic pregnancy, Machine learning

## Abstract

**Objective:**

To evaluate the accuracy of neural networks and Naïve Bayes models in diagnosing ectopic pregnancy, using clinical data, hCG levels, and transvaginal ultrasound findings from a real dataset.

**Methods:**

This was a retrospective cohort study based on a public dataset of 2,495 first-trimester pregnant women with confirmed pregnancy under 13 weeks, documented transvaginal ultrasound reports, and follow-up on pregnancy outcome. The cohort presented a natural imbalance (8.5% ectopic, 91.5% intrauterine pregnancies), reflecting real-world clinical prevalence. Data on risk factors, clinical symptoms, ultrasound findings, and serial hCG levels were included. The dataset was preprocessed and split into training (80%) and testing (20%) sets using stratified sampling based on pregnancy outcome to preserve the proportion of ectopic cases in both sets. The main outcome measures were accuracy, sensitivity, specificity, and F1 score.

**Results:**

The neural network model achieved an accuracy of 99.4%, sensitivity of 94.6%, specificity of 97.2%, and an F1 score of 95.9%. The Naïve Bayes model showed an accuracy of 96.5%, sensitivity of 98.1%, specificity of 71.2%, and an F1 score of 82.5%. Both models were validated without evidence of overfitting.

**Conclusion:**

The neural network model demonstrated statistically significant superior accuracy and reliability in diagnosing ectopic pregnancy compared to the Naïve Bayes model (McNemar's test, p < 0.001), suggesting the potential of machine learning models, particularly deep learning, to enhance early diagnosis and clinical decision-making.

## Introduction

Ectopic pregnancy (EP) is a critical condition in obstetrics, occurring in approximately 2% of all pregnancies, but with a higher incidence in emergency settings, ranging from 6% to 16%.^(1,2)^ The timely and accurate diagnosis of EP is essential to prevent severe complications, including tubal rupture and hemorrhage, which can be life-threatening.^(3)^ Traditional diagnostic methods primarily rely on the measurement of serum human chorionic gonadotropin (hCG) levels and transvaginal ultrasound (TVUS).^(4)^ However, these methods can sometimes be inconclusive, necessitating the development of more sophisticated diagnostic tools.

Machine learning (ML) has emerged as a powerful tool in medical diagnostics, offering the potential to improve the accuracy and efficiency of disease detection.^(5)^ Among the various ML techniques, neural networks and Naive Bayes models have shown significant promise. Neural networks, with their ability to model complex, non-linear relationships, can learn from large datasets and identify patterns that may not be apparent through traditional statistical methods.^(6)^ Naive Bayes, a probabilistic classifier based on Bayes’ theorem, is particularly effective in scenarios where the assumption of feature independence holds, providing robust performance with relatively simple computational requirements.^(7)^ Our group has demonstrated the utility of the use of an algorithm based on Bayes’ theorem in diagnosing EP by integrating clinical signs, symptoms, risk factors, TVUS findings, and hCG levels.^(8)^ However, it would be of interest to involve the division of data into training and test sets to provide a more robust result. Addressing this methodology is crucial to enhance the reliability and applicability of ML models in clinical practice, since overfitting can reduce generalizability of the model, as it does not adequately assess the model's performance on unseen data.^(9)^

In this study, we aim to evaluate the diagnostic performance of neural networks and Naïve Bayes models in predicting EP. We will employ a robust methodology by splitting our dataset into training and test sets, ensuring that the models are validated on independent data. This approach will allow us to compare the diagnostic accuracy, sensitivity, and specificity of these models in a real-world clinical setting.

Our dataset will consist of first-trimester pregnant women presenting at a gynecological emergency unit. We will include a comprehensive array of clinical and diagnostic variables, such as patient history, clinical symptoms, TVUS findings, and hCG levels. By leveraging the strengths of neural networks and Naïve Bayes models, we aim to develop a more accurate and reliable diagnostic tool for EP.

The integration of advanced ML techniques in the diagnostic process aligns with the current trends in precision medicine, aiming to provide clinicians with more reliable tools for decision-making.

## Methods

This study had an exemption from the institutional review board for using a publicly available dataset from Harvard Dataverse.^(10)^ We used the public and anonymized dataset from our previous study for analysis.^(8,10)^ The dataset comprises 2495 entries, considering the clinical and diagnostic parameters associated with ectopic pregnancy (features). The features in the dataset include risk factors, symptoms and signs recorded during each consultation, transvaginal ultrasound findings, hCG levels measured at different times, and the final binary outcome indicating the presence or absence of an ectopic pregnancy (to access the file, use the link https://github.com/tuxcuiabano/EctopicPregnancy). These features were chosen due to their relevance in diagnosing ectopic pregnancies and availability in the clinical dataset. Details of these features are in our original work.^(8)^

The final dataset consisted of 2,495 patients. Inclusion criteria were: first-trimester pregnant women with suspected ectopic pregnancy, who underwent transvaginal ultrasound and had confirmatory follow-up data. Patients with incomplete data on pregnancy outcomes were excluded. This real cohort presented a natural imbalance, with 8.5% (n=212) of ectopic pregnancy cases and 91.5% (n=2,283) of intrauterine pregnancies, which reflects daily clinical practice. The implications of this imbalance for classification metrics were discussed in the limitations section.

Several preprocessing steps were undertaken to prepare the data for analysis before submitting the dataset to Keras library. Initially, the dataset was loaded into a data frame for inspection and handling of missing values. Any missing values in the dataset were identified and appropriately addressed to avoid introducing bias or inaccuracies into the model. Analysis of our dataset revealed that missing data were predominantly structural rather than random, reflecting the longitudinal nature of the clinical cohort. All variables from the initial consultation (baseline) were 100% complete for all 2,495 patients. Missing data in subsequent consultations (consultations 2 through 7) represent the expected clinical pattern where not all patients require multiple follow-up visits after initial evaluation. Specifically, only 19.7% of patients returned for a second consultation, and 4.7% for a third consultation, indicating that diagnosis was frequently established during the initial visits. This pattern of missing data is characteristic of real-world clinical practice and does not represent a methodological limitation. For numerical columns, missing values were imputed with the mean, ensuring that the overall distribution of the data remained consistent. For categorical columns, the most frequent value was used for imputation, maintaining the integrity of the categorical distribution.

Categorical variables were then encoded using one-hot encoding. This technique converts categorical variables into a format suitable for machine learning algorithms by creating binary columns for each category. This step was crucial for ensuring the proposed neural network could effectively utilize the categorical data. Following the encoding process, feature scaling was applied to the numerical features. Standardization was chosen to scale the data, resulting in each feature having a mean of zero and a standard deviation of one. This normalization process is essential for neural networks as it ensures that all input features contribute equally to the learning process and help achieve faster convergence during training.

For the preprocessing to Orange 3, eight features were removed from the original 31 features (columns). The remaining features were the binary outcome (ectopic, no ectopic), which was the target feature, while the presence of risk factors (yes/no), signs and symptoms in consultations 1 to 7, ultrasound findings in consultations 1 to 7, hCG levels from consultation 1 to 7 were considered as categorical features. The difference in percentage of hCG levels between consultation 1 and 2 was considered as a numerical feature. The remaining preprocessing was performed as the default setting in Orange 3, in the following order: 1) instances with unknown target values were removed, 2) one-hot-encoding for categorical values, 3) empty columns were removed, and 4) missing values were imputed with mean values normalizes the data by centering to mean and scaling to standard deviation of 1.

**Figure 1:**
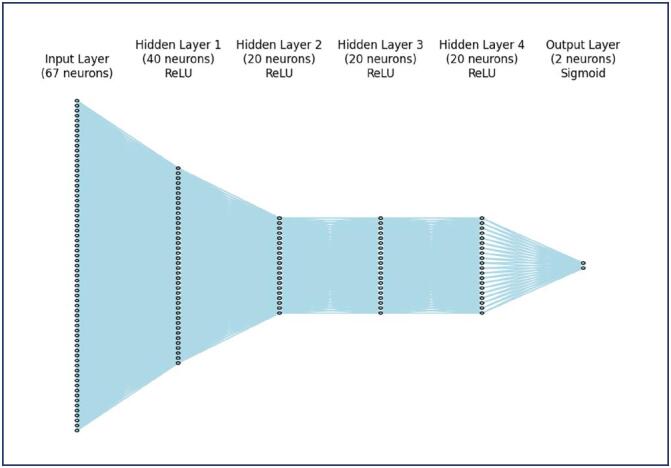
Structure of the Neural Network

The dataset was divided into training (80%) and testing (20%) sets using stratified sampling based on the outcome (ectopic pregnancy vs. non-ectopic pregnancy). This procedure ensured that the proportion of cases in each class was preserved in both sets, preventing sampling bias. By partitioning the data in this manner, the model was trained on 80% of the data, while the remaining 20% was reserved for evaluating its performance. This approach helps assess the generalization capability of the model on unseen data. The neural network model was designed using the Keras library, incorporating multiple layers to capture the complexity of the data. The input layer was configured with 67 neurons, corresponding to the number of features in the preprocessed dataset. The first dense layer consisted of 40 neurons, utilizing the Rectified Linear Unit (ReLU) activation function to introduce non-linearity and enable the model to learn complex patterns. The architecture included three intermediate dense layers with 20 neurons and ReLU activation. These layers were crucial for capturing deeper representations of the data. The output layer was designed with two neurons and a sigmoid activation function, which is suitable for the binary classification task of identifying ectopic pregnancies. Figure 1 shows the structure of the designed neural network.

The model training involved an extensive process to optimize the neural network parameters. The network was trained over 200 epochs, with a batch size of 350, ensuring that the model had sufficient exposure to the data to learn effectively. The Adam optimizer, known for its efficiency and adaptive learning rate capabilities, was used with a learning rate of 0.001. The training process involved forward propagation, loss calculation, backpropagation, weight updates, and iteratively refining the model parameters to minimize the loss function.

The neural network architecture was implemented using the categorical cross-entropy loss function, suitable for the one-hot encoded binary classification task. Overfitting was monitored by observing the validation accuracy curve, which did not show significant divergence from the training accuracy. The total number of trainable parameters in the model was 4,422. No explicit regularization techniques, such as L2 or Dropout, were employed as the model demonstrated good generalization on the test set. The full Python script is available at https://github.com/tuxcuiabano/EctopicPregnancy.

For an alternative analysis of the original dataset, we utilized Orange 3, an open-source, Python-based data mining and visualization platform. This software offers a user-friendly graphical user interface that allows users to perform data analysis and visualization without requiring extensive programming knowledge.^(11)^ Specifically, we employed the neural network and Naïve Bayes algorithms available in Orange 3 to analyze the dataset. For the neural network, the following parameters were used: 169 layers of neurons, ReLU as activation method, Adam as solver, and a stratified 10 fold cross validation, using a learning rate of 0.001 and a maximal number of iterations of 350.

The requirement for ethical approval was waived because the study involved only publicly available, de-identified data.

## Results

**Figure 2:**
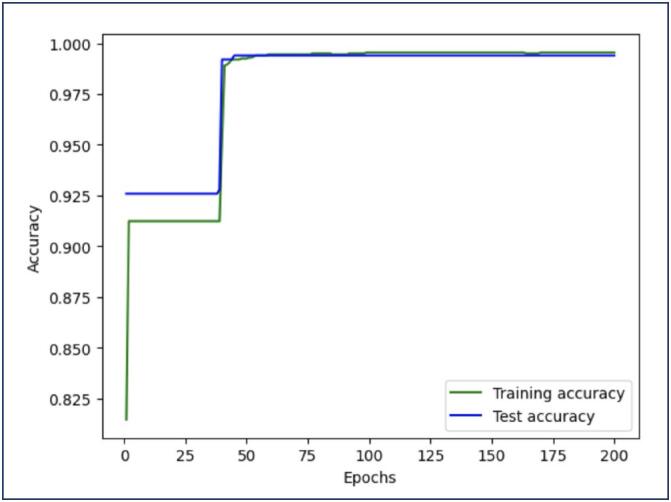
Performance of the model. The x-axis represents the number of epochs, ranging from 0 to 200, while the y-axis indicates the model's accuracy, measured as a percentage. The graph includes two lines: one representing the training accuracy and the other representing the test accuracy, allowing us to assess both the model's performance on the training data and its generalization to unseen test data

The graph presented in figure 2 depicts the relationship between epochs and accuracy, providing a detailed view of the neural network's learning process over the course of training. The performance of the model had an accuracy (CA) of 99.4% (Table 1). Further details can be seen in the confusion matrix (Table 2).

**Table 1:** Performance (%) of Naive Bayes and Neural Network targeting cases of ectopic pregnancy, using Orange 3 (O) and Keras (K)

Model	AUC	CA	F1 Score	Precision	Recall
(O)Naive Bayes	99 [98.6-99.4]	96.5 [95.7-97.2]	82.5 [81.1-84]	71.2 [66-76.4]	98.1 [96.3-99.9]
(O)Neural Network	99.1 [98.7-99.4]	98.7 [98.2-99.1]	92.1 [91.1-93.2]	93.2 [89.8-96.7]	91 [87.2-94.9]
(K)Neural Network	99.8 [98.6 - 99.9	99.4 [98.2 - 99.8]	95.9% [93.7-97.2]	97.2 [95.3-98.3]	94.6 [92.2-96.3]

AUC: area under the curve, CA: Classification Accuracy, F1 Score: It is the harmonic mean of precision and recall, providing a single metric that balances both concerns. Precision: (Positive predictive value) Precision measures the proportion of true positive results among all positive predictions made by the model. A high precision score indicates that the model has a low rate of false positives. Recall:(Sensitivity): Recall measures the proportion of true positive results among all actual positive cases. Numbers in [ ] are 95% confidence intervals

**Table 2:** Confusion matrix of the number of instances in the Neural Network and Naïves Bayes. The performance of models developed in Orange 3 (using 10-fold cross-validation) and the Keras-based neural network are presented herein. The Keras model results reflect its performance on the independent test set (20% of the data), which was not used during training. This test set evaluation is a standard practice to assess the model's ability to generalize to new, unseen data

		Predicted	
		No ectopic	Ectopic
Naïves Bayes (Orange 3)			
Actual	No ectopic	2199	84
	Ectopic	4	208
Neural Network (Orange 3)			
Actual	No ectopic	2269	14
	Ectopic	19	193
Neural Network (Keras library - test 20%)			
Actual	No ectopic	461	1
	Ectopic	2	35

In Orange 3, the results from neural network and Naive Bayes models resulted in a similar accuracy: 98.7% and 96.5%, respectively, as shown in table 1. Individual number instances from the confusion matrix for Naïves Bayes and Neural Network are in table 2.

Table 2 presents the confusion matrices for three model evaluations: Naive Bayes (Orange 3), Neural Network (Orange 3), and Neural Network (Keras library - test 20%). The Keras model results specifically reflect its performance on the independent test set (20% of the data), which was held out and not used during training. This test set evaluation serves as an independent validation of the model's generalization capability on unseen data, a standard practice to assess the robustness of machine learning models.

## Discussion

We aimed to develop and evaluate a neural network model and a Naïve Bayes model for accurately classifying ectopic pregnancies based on clinical and diagnostic parameters from our previous publications.^(8,10)^ By using these models in ML, we addressed questions about overfitting. Overfitting would be indicated by a significant divergence between training and test accuracies, where the training accuracy continues to improve while the test accuracy declines. However, in our experiments, both accuracies stabilize and remain close, suggesting that the model has not overfitted and retains good generalization capability. However, in our experiments, both accuracies stabilize and remain close, suggesting that the model has not overfitted and retains good generalization capability.

The experiments demonstrated the neural network's high efficacy in learning from the data and generalizing it to unseen cases. The model's accuracy is relatively low at the onset of training, particularly for the training set. This initial low accuracy is expected, as the model starts with random weights and has not yet learned from the data. The training accuracy started and quickly moved to 0.9 within the initial epochs, indicating the model's initial adjustments to the data. The test accuracy starts slightly higher than the training accuracy, indicating that the initial random weights fit the test data slightly better. The object-oriented software (Orange 3) had a similar performance, using the 10-fold cross-validation, either in the Naïves Bayes or in the Neural Network models.

A crucial point observed was the convergence of training and test precision lines around the 25th epoch. At this point, the model reaches a state where it effectively captures the patterns in the data without overfitting them. This convergence is a positive indicator that the model is balanced, avoiding the problem of overfitting. The stability observed in the precision curves indicates that the model can generalize well to new data, not just memorizing the training data but capturing underlying patterns. This suggests that the model is well-trained, being able to maintain its performance even when confronted with previously unseen data.^(9)^

Our results are better than those published by Rueangket et al.^(12)^ Possible explanations for these differences can be related to the features used in both models. Rueangket et al.^(12)^ did not use a threshold of 2,000 mUI/ml for hCG; instead, they used 1,000 mIU/mL. In addition, they used multiparity as a feature, while we did not.

The neural network architecture implemented in our study was carefully designed to capture the complexity of clinical data related to ectopic pregnancy. The input layer was configured with 67 neurons, corresponding to the number of features in the preprocessed dataset. The first dense layer consisted of 40 neurons, utilizing the Rectified Linear Unit (ReLU) activation function to introduce non-linearity and enable the model to learn complex patterns. The architecture included three intermediate dense layers with 20 neurons and ReLU activation. These layers were crucial for capturing deeper representations of the data. The output layer was designed with two neurons and a sigmoid activation function, which is suitable for the binary classification task of identifying ectopic pregnancies.^(7)^

Comparing our model with other recent studies, we observed that different machine learning approaches have been explored for the diagnosis of ectopic pregnancy. For example, Rueangket et al.^(12)^ used a combination of logistic regression, support vector machines, decision trees, and neural networks, achieving a maximum accuracy of 87.9% with logistic regression.^(12)^ In contrast, our Keras-based neural network achieved an accuracy of 99.4%. To statistically compare the accuracy of the models, we applied McNemar's test. The result indicated that the neural network's performance was significantly superior to the Orange Naive Bayes model (χ² = 53.02, p < 0.001), validating the observation of numerical superiority.

A 2025 study introduced a machine learning-based diagnostic model configured with modified balancing weight optimization (MBWO), achieving an accuracy of 98%.^(13)^ This result is comparable to our neural network model, which achieved 99.4% accuracy, suggesting that different machine learning architectures can be effective for this clinical application.

Additionally, researchers developed PROMETHEUS (Prediction of Methotrexate for Ectopic Pregnancy Treatment Success), an AI-based decision support tool for predicting the success of methotrexate treatment in ectopic pregnancies.^(14)^ While our model focuses on diagnosis, PROMETHEUS addresses the subsequent stage of therapeutic management, highlighting the potential of machine learning across the entire spectrum of care for ectopic pregnancy. A recent systematic review on artificial intelligence (AI)-enhanced clinical decision support systems in obstetric care highlighted that, following an ectopic pregnancy, machine learning-based clinical decision support systems have been explored to help patients and healthcare professionals make more informed decisions.^(15)^ This underscores the growing importance and acceptance of AI-based approaches in the management of ectopic pregnancy.

This detailed methodology underscores the comprehensive steps to ensure the dataset was adequately preprocessed and the neural network model was effectively trained. By meticulously handling missing values, encoding categorical variables, scaling features, and employing a robust neural network architecture, we were able to develop a highly accurate and reliable model.

In the neural network, the F1 score of >92%, which balances precision and recall, indicates that the model maintains a good trade-off between these two metrics, providing a reliable measure of its overall performance. This balance is essential, as it ensures that the model accurately identifies positive cases and consistently does so without favoring one metric over the other. These metrics underscore the effectiveness of the neural network in handling the binary classification task, ensuring reliable and accurate outcomes. We reproduced these results using different approaches, i.e., Orange 3. We also provided the dataset and Python's code for analysis and reproduction.

An important aspect of our model is its generalization capability. The analysis of training and test accuracy curves demonstrates that the model not only effectively learns from the training data but also maintains high performance when applied to unseen data. This characteristic is crucial for clinical applications, where the model must be able to make accurate predictions in new patients with varying presentations.^(7)^

Furthermore, our validation approach, using both internal and external validation, provides a more robust assessment of model performance compared to studies that use only one validation method. The 10-fold crossvalidation implemented in Orange 3 complements the 80-20% train-test split used in the Keras library, offering a more comprehensive view of the model's stability and reliability.^(6)^

The implementation of different machine learning frameworks (Keras and Orange 3) to develop and validate our models also represents a methodological strength. This dual approach allows for direct comparison of different implementations and confirms the robustness of the results, regardless of the platform used.^(12)^

The limitations of this study are related to the dataset and they reduce external validity, e.g. the use of single hCG kit and all data are derived from a single center.^(8)^ In addition, Keras results are derived from one seed. Due to the random nature of the machine learning process, results vary within a certain range. Herein, we set a random seed in the Python code to maintain consistency of the results.

Another potential limitation is the need for prospective validation in diverse clinical settings. Although our model has demonstrated excellent performance on the available data, its applicability across different populations, clinical settings, and diagnostic protocols still needs to be evaluated.^(3)^ The successful implementation of machine learning models in clinical practice requires not only technical accuracy but also acceptance by healthcare professionals and integration into existing workflows.

Additionally, while we used a comprehensive set of clinical and diagnostic features, there are other potentially relevant factors that were not included in our model. Genetic factors, emerging biomarkers, and detailed sociodemographic characteristics could potentially further improve the model's performance and clinical applicability.^(4)^

There are some perspectives. Based on our models, new cases can be tested, using the friendlier widget "Predictions" from the Orange 3 software. Eventually, an application with data sharing would be possible to validate the model on a large scale.

An important direction for future work involves the integration of the model into clinical workflows. Conducting prospective validation studies in real-world clinical settings would help assess the model's practical utility and robustness. Moreover, developing user-friendly interfaces and decision support tools based on the neural network could facilitate its adoption by healthcare providers, ultimately enhancing the accuracy and efficiency of ectopic pregnancy diagnosis.^(2)^

Another promising area for future research is the exploration of more advanced deep learning techniques, such as convolutional neural networks (CNNs) for ultrasound image analysis, or reinforcement learning models for optimizing treatment strategies. The integration of multiple data modalities, including clinical, laboratory, genomic, and imaging data, could potentially further improve diagnostic accuracy and provide more comprehensive insights into the pathophysiology of ectopic pregnancy.^(1)^ For instance, Suresh et al.^(13)^ reported an automated ectopic pregnancy prediction system using ultrasound images with the aid of a deep learning technique, employing an improved spiking neural network with a seagull optimization algorithm (ISNN-SOA).^(13)^ This approach represents a significant advancement in integrating image analysis with machine learning for ectopic pregnancy diagnosis, complementing our work that primarily focuses on clinical and laboratory data.

Furthermore, the application of model interpretability techniques, such as SHAP (SHapley Additive exPlanations) or LIME (Local Interpretable Model-agnostic Explanations), could help elucidate the most influential factors in the model's predictions, potentially revealing new biomarkers or risk factors for ectopic pregnancy.^(15)^ This approach would not only enhance transparency and trust in the model but could also contribute to advances in medical knowledge about this condition.

Finally, ongoing monitoring and updating of the model with new data will be essential to maintain its relevance and effectiveness in a constantly evolving clinical landscape. Implementing continuous learning strategies would allow the model to adapt to changes in clinical practices, patient profiles, and diagnostic technologies over time.^(16)^

## Conclusion

In conclusion, our study demonstrates that the neural network model provides a highly accurate and robust method for diagnosing ectopic pregnancy within our dataset, outperforming the Naïve Bayes model with statistical significance. The primary contribution of this work is the development of a reliable machine learning tool that shows strong potential for clinical application. However, the critical next step is to move beyond retrospective data and conduct prospective validation studies to confirm the model's effectiveness and safety in real-world clinical settings before it can be recommended for widespread adoption.

## Data Availability

The research data are described in the article presented.
